# Polyurethane/Polylactide-Blend Films Doped with Zinc Ions for the Growth and Expansion of Human Olfactory Ensheathing Cells (OECs) and Adipose-Derived Mesenchymal Stromal Stem Cells (ASCs) for Regenerative Medicine Applications

**DOI:** 10.3390/polym8050175

**Published:** 2016-04-30

**Authors:** Krzysztof Marycz, Monika Marędziak, Jakub Grzesiak, Dariusz Szarek, Anna Lis, Jadwiga Laska

**Affiliations:** 1 Electron Microscopy Laboratory, Wroclaw University of Environmental and Life Sciences, Wroclaw 51-631, Poland; 2 Department of Animal Physiology and Biostructure, Wroclaw University of Environmental and Life Sciences, Wroclaw 50-375, Poland; monika.maredziak@gmail.com; 3 Electron Microscopy Laboratory, Wroclaw Research Centre EIT+, Wroclaw 54-066, Poland; grzesiak.kuba@gmail.com; 4 Department of Neurosurgery, Lower Silesia Specialist Hospital of T. Marciniak, Emergency Medicine Centre, Wroclaw 54-049, Poland; szarekdariusz@gmail.com; 5 Faculty of Materials Science and Ceramics, AGH University of Science and Technology, Krakow 30-059, Poland; annal@agh.edu.pl (A.L.); jlaska@agh.edu.pl (J.L.)

**Keywords:** polyurethane, polylactide, zinc oxide, adipose stromal stem cells, olfactory ensheathing cells

## Abstract

Polymeric biomaterials based on polyurethane and polylactide blends are promising candidates for regenerative medicine applications as biocompatible, bioresorbable carriers. In current research we showed that 80/20 polyurethane/polylactide blends (PU/PLDL) with confirmed biological properties *in vitro* may be further improved by the addition of ZnO nanoparticles for the delivery of bioactive zinc oxide for cells. The PU/PLDL blends were doped with different concentrations of ZnO (0.001%, 0.01%, 0.05%) and undertaken for *in vitro* biological evaluation using human adipose stromal stem cells (ASCs) and olfactory ensheathing cells (OECs). The addition of 0.001% of ZnO to the biomaterials positively influenced the morphology, proliferation, and phenotype of cells cultured on the scaffolds. Moreover, the analysis of oxidative stress markers revealed that 0.001% of ZnO added to the material decreased the stress level in both cell lines. In addition, the levels of neural-specific genes were upregulated in OECs when cultured on sample 0.001 ZnO, while the apoptosis-related genes were downregulated in OECs and ASCs in the same group. Therefore, we showed that PU/PLDL blends doped with 0.001% of ZnO exert beneficial influence on ASCs and OECs *in vitro* and they may be considered for future applications in the field of regenerative medicine.

## 1. Introduction

The limited regenerative ability of injured central nervous system (CNS), including its mechanical and neurodegenerative failures, becomes a real challenge for advanced regenerative medicine [1]. Various strategies has been proposed to support regeneration of spinal cord, however, application of a specific biomaterial combined with living cells seems to be the most promising [2,3]. Numerous published data confirm improved neural regenerative capability of the spinal cord supported with a biomaterial compared to the non-treated one [4,5]. Polymer biomaterials are widely used in manufacturing of modern biomedical devices and/or implants. They also seem highly acceptable and promising for spinal cord injuries (SCI) treatment, mostly because of their low immunogenicity, biodegradability, and biocompatibility [6]. Various polymers, including polyurethane (PU) and polylactide (PLDL), has been widely investigated for neuroregenerative purposes [7,8]. Polylactide is known for low cytotoxicity of its degradation products; however, at the same time, it exhibits limited biomechanical characteristics and might decrease the pH due to the release of lactic acid during degradation [9]. Recently, the blends of the above mentioned polymers *i.e.*, PU/PLDL have been shown to possess improved mechanical properties and thus being more promising materials not only from a surgical perspective, but also for culture and delivery of regenerative cell populations [9,10]. Changes in other physicochemical properties of the polymer material, like wettability and roughness, depending on the relative content of the two polymers have also been observed. It was shown in our previous study that PU/PLDL with a weight ratio of 80:20, shows elevated Young’s modulus, improved wettability, and higher roughness—crucial factors for the attachment and survival of cellular components [9]. We also proved that the material is suitable for SCI treatment, however, further improvement of its biocompatibility or bioactivity is required to support growth, survival, and the anti-oxidative defense of cells used for SCI regeneration.

Evidence-based clinical studies are intensively focused on the application of stem cell-based therapy and/or olfactory derived glial cells (OECs) in the treatment of SCI [11,12]. Recently, transplantation OECs has been shown to possess neuroregenerative capacity in treatment of patients suffering from total SCI [13]. In turn, adipose-derived mesenchymal stromal stem cells (ASCs) are considered serious candidates because of their ability to differentiate toward the glial lineage [14]. ASCs has been reported to possess ability to prevent regenerating axons of apoptosis, and replace lost cells, particularly oligodendrocytes, in order to facilitate the remyelination of spared axons [15]. In a more recent study, ASCs transplanted in a three-dimensional cell mass (3DCM-ASCs) in hind limb model caused enhanced axonal outgrowth and elevated density of neovascular formations [16]. These facts brought a promising possibility for the use of ASCs seeded onto biomaterial for SCI treatment.

For many years, zinc has been shown to play an important role in central nervous system as a neurosecretory factor, which is highly concentrated in the synaptic vesicles, within the so-called “zinc-containing” neurons [17]. Zinc, as a biofactor, is responsible for activation of numerous enzymes engaged in the metabolic processes and simultaneously in brain development from the early neonatal stage to the maintenance of brain function in adults [18]. Moreover, zinc has been shown to modulate synaptic activity and neural plasticity in young and elderly patients. Zinc has also been shown as an anti-oxidative defense in a dose-dependent manner, which might be essential in the course of regenerative processes of the CNS, especially when a combination of particular biomaterials with cellular compartments is considered [19]. The stress factors, including reactive oxygen species (ROS), nitric oxide (NO,) and free radicals scavengers, like superoxide dismutase (SOD), has been shown to play an important role in cytophysiology of both mesenchymal stromal stem cells as well as glial cells [20,21]. An imbalance between free radicals and antioxidants has been reported as an essential factor that affects MSCs’ multipotent character through activation of particular gene expression, proliferation, and differentiation. Predominance of oxidants over antioxidants leads to initiation of the apoptosis process through upregulation of the p53 gene (tumor suppressor), changes in the expression of pro- and anti-apoptotic Bcl-2 family members, cytochrome C relocation, activation of caspases, chromatin condensation, and DNA fragmentation [22].

In our previous findings, we showed that PU/PLDL based biomaterials can be satisfactorily used for scaffold fabrication for both rat olfactory ensheathing cells (OECs) as well as rat adipose-derived mesenchymal stromal stem cells (ASCs). In this study, we doped PU/PLDL blends with different amounts of zinc ions to increase its bioactivity, and in consequence, viability and activity of human-derived ASCs and OECs. We have found that even the lowest concentration of zinc incorporated into the investigated biomaterial significantly decreased the impact of oxidative factors and induced activity of free radical scavengers.

## 2. Materials and Methods

### 2.1. Materials

The reactants used were zinc oxide (Sigma-Aldrich, Poznan, Poland, puriss. p.a., ACS reagent, Nijmegen, Netherlands, ≥99.0%), *N*,*N*-Dimethylformamide(DMF) as a solvent (POCH S.A., Gliwice, Poland, pure), and sterile-filtered water for molecular biology and cell culture (Sigma-Aldrich, Poznan, Poland). The biodegradable polymers used in our study were rigid poly(l-lactide-*co*-d,l-lactide) (PLDL) with a molar ratio l-lactide to d,l-lactide of 80:20 (PURAC biochembv., Gorinchem, The Netherlands), and elastomeric polyurethane (Bayer, Leverkusen, Germany). Zinc oxide in the form of nanopowder, particle size <100 nm, was purchased from Sigma-Aldrich.

### 2.2. Preparation of Biomaterials

**Scheme 1:**
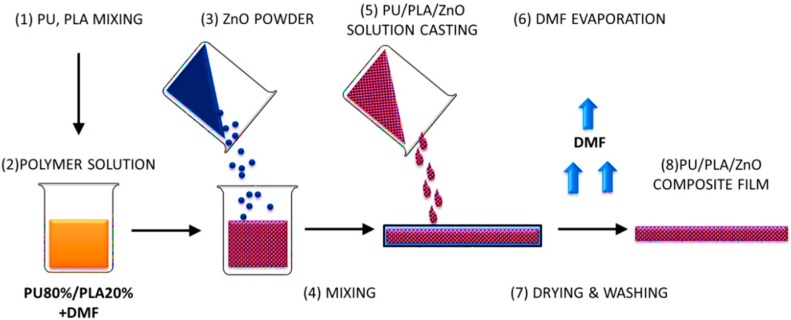
Schematic illustration of the solvent casting method used for PU/PLA/ZnO composite fabrication.

The polymer composite films were prepared via solvent casting technique (Scheme 1). First, the polymer solutions with the polymer concentration of 5% were prepared. Polymer granules (PLA and PU 20/80 *w*/*w*) were dissolved in DMF by magnetic stirring (MS11H, Pruszkow, Poland) for three days at 40 °C. The PU/PLA solution was then mixed with ZnO nanopowder to obtain different concentrations. The zinc oxide concentrations in the PU/PLA solutions were 0.001, 0.01, and 0.05 wt %. The resulting mixture was stirred with a magnetic stirrer at 40 °C for 24 h to obtain a uniform suspension. Then the solution was stirred with an ultrasonic homogenizer for 15 min before casting. The prepared PU/PLA/ZnO mixture was cast into a glass Petri dish. After DMF evaporation in vacuum (2 × 24 h) (SPU-200 vacuum drier) at 25°–30°, the composite films were removed from the glass molds. The PU/PLA/ZnO films were rinsed with filtered distilled water and dried in air and vacuum at room temperature, and then sterilized with cold H_2_O_2_ plasma. Sterile packages (EN 868/ISO 11607, Sigma-Aldrich, Poznan, Poland) were arranged.

### 2.3. Morphology of the PU/PLDL/ZnO Films

Morphology of the PU/PLDL/ZnO composite films was examined by scanning electron microscopy (SEM) (EVO LS15, Zeiss, Oberkochen, Germany). Before observation, the samples were coated with gold using a sputter coater. The distribution of ZnO incorporated within the PU/PLDL 80:20 blends was studied by carrying out SEM-EDX mapping (Bruker, Billerica, MA, USA).

### 2.4. Water Contact Angle Measurement

Surface wettability is a crucial property for biomaterials/scaffolds intended for adherent cell culture. Wettability of the PU/PLDL/ZnO composite films was determined by the measurements of the static contact angles of distilled water drops on the surface of the films using a contact angle goniometer (DSA 10, Krüss, Hamburg, Germany). Measurements were performed at room temperature, using 2 µL water drops deposited with a micro-syringe. The values of the static contact angle were obtained as an average of the values of ten measurements for each composite sample.

### 2.5. Atomic Force Microscopy

Atomic force microscopy (AFM Explorer, Veeco, Oyster Bay, NY, USA) studies were performed. Changes in surface morphology are quantified by roughness values (*R*q) for the top and bottom sides of prepared PU/PLDL based films. The roughness value is defined as follows:

$$Rq=\;\sqrt{\frac{ZC-ZA}{N}}$$

where *Z*_A_ is the average *Z* height value within a given area, *Z*_C_ is the current *Z* value and *N* is the number of points within the given area. Possible significant differences among groups were studied with the Student's *t*-test. Statistical significance was considered at a probability of *p* < 0.05.

### 2.6. Cell Isolation

The experiment was approved by the Local Bioethics Committee of Wroclaw Medical School (registry number KB-177/2014, March 2014). Olfactory ensheathing glial cells were isolated according to the protocol described previously [23]. Briefly, human olfactory bulbs were dissected from brains *post mortem* from four dead donors: (1) 28 years old, ~6 h post mortem; (2) 36 years old, ~12 h post mortem; (3) 51 years old, ~12 h post mortem; (4) 46 years old, ~17 h post mortem. After the collection, tissues were placed in sterile Hank’s Balanced Salt Solution (HBSS, Sigma-Aldrich) for transportation. Bulbs were carefully placed under the class II safety cabinet, and the meninges were cut using fine scissors. Next, meninges were removed by rolling the tissue on sterile filter paper. Subsequently, tissues were gently cut on smaller pieces, washed in HBSS, and placed in an enzyme solution (general type collagenase in DMEM/F12 Ham’s, 5 mg/mL, Sigma) for 10 min at 37 °C. After the incubation, the enzyme was deactivated by the addition of 10% fetal bovine serum (FBS, Sigma). Tissues were then disrupted using syringe needles of gradually decreasing size (18 G, 20 G, 22 G, 24G), followed by their centrifugation at 300× *g* for 10 min. Subsequently, supernatants were discarded, and the cells were washed in fresh HBSS. After another centrifugation, cells were disrupted using 40 µm cell strainer (BD Science). Cells were suspended in fresh DMEM/F12 Ham’s containing the 10% of FBS and 1% of penicillin/streptomycin/amphotericin b solution (P/S/A) (all from Sigma). Cells were cultured in T-25 culture flasks at 37 °C/5% CO_2_ in humidified incubator for five days without disturbing. At the day five, when cells achieved the proper adhesion and morphology, they were detached from culture surface using TrypLE select (Thermo Scientific, Waltham, MA, USA), and undertaken for experiment. For this purpose, 3 × 10^4^ cells were seeded on biomaterials placed in a 24-well plate, and maintained in DMEM/F12 Ham’s with 10% FBS and 1% of P/S/A. As a control, cells were seeded on biomaterial without the addition of ZnO.

Adipose-derived mesenchymal stromal stem cells were isolated using the protocol described previously [24]. Subcutaneous adipose tissue was donated by the four patients undergoing the orthopedic prosthesis implantation: (1) 70 years old; (2) 47 years old; (3) 68 years old; and (4) 70 years old. Tissues were collected from the region of the hip joint and placed in sterile HBSS for transportation. For cell isolation, tissues were washed extensively in HBSS, followed by their fine mincing using surgical blade. Chopped tissues were then placed in collagenase type I solution (5 mg/mL in DMEM, Sigma) for 40 min at 37 °C with strong shaking every 10 min. After the digestion, solution was centrifuged at 1200× *g* for 10 min to separate cells from the enzyme solution, remaining tissue and lipids released from the adipocytes. The supernatants were discarded, and the cells were resuspended in DMEM/F12 Ham’s containing 10% of FBS and 1% of P/S/A. Cells were propagated in T-25 culture flasks at 37 °C/5% CO_2_ in humidified incubator for five days, until they achieved the full confluence. Subsequently, cells were passaged once using TrypLE select (Thermo Scientific), and further maintained in DMEM containing 4500 mg/L of glucose, 10% of FBS and 1% of P/S/A, before they were used for experiment. For this purpose, 3 × 10^4^ cells were seeded on biomaterials placed in 24-well plate, and cultured in DMEM (4500 mg/L glucose, 10% FBS, 1% P/S/A). As a control, cells were seeded on biomaterial without the addition of ZnO.

### 2.7. Cell Viability and Proliferation

The viability and proliferative activity of cells cultured on biomaterials and without the biomaterial were evaluated using a resazurin-based indicator of cell metabolic activity (Alamar Blue, Sigma). Measurements were performed at day one, two, and five in both cell lines. For experiment, cell media were collected and replaced with fresh medium containing 10% of resazurin dye. Cultures were incubated for 2 h at 37 °C, followed by the collection of media. Collected media were undertaken for spectrophotometric measurements by means of microplate reader (BMG Labtech, Ortenberg, Germany). Absorbance was measured at 600 nm, with the subtraction of plate background absorbance at 690 nm. A decrease in absorbance in all analyzed groups, in relation to not incubated blank sample, was compared to the standard curve to determine viable cell numbers. These values were used for the calculation of the population doubling time parameter, using an algorithm available online [25]. After the incubation, cells on materials and in the control were fixed in 4% paraformaldehyde for fluorescence microscopy or in 2.5% glutaraldehyde for electron microscopy.

### 2.8. OEC Phenotype

The proper p75+/GFAP+ phenotype of olfactory ensheathing cells was verified at day five of the culture on the biomaterials. For immunofluorescence staining, cells were fixed in 4% paraformaldehyde for 15 min at room temp. After fixation, cells were washed three times in PBS, and permeabilized using 0.05% triton x-100 solution in PBS for 20 min. After triple washing in PBS, cells were incubated with primary antibodies (anti-GFAP produced in rabbit, ab7260, 1:1000; anti-p75 NGF receptor produced in rabbit, ab8874, 1:1000; all from Abcam, Cambridge, UK) for one hour at 37 °C. After triple washing in PBS containing 2% of FBS, cells were incubated with secondary antibodies (goat anti-rabbit IgG conjugated to atto-488 fluorophore, Sigma, 1:400) for one hour at 37 °C. After incubation, cells were washed three times in PBS and counterstained with 4',6-diamidino-2-phenylindole (DAPI, Sigma-Aldrich, 5 µg/mL) for 5 min. Preparations were observed by means of inverted fluorescence microscope (AxioObserver A1, Zeiss), and documented with a digital camera (PowerShotA630, Canon, Tokyo, Japan). Pictures were merged using ImageJ software.

### 2.9. Scanning Electron Microscopy

Cells were fixed in 2.5% glutaraldehyde, followed by their triple rinsing in phosphate buffer (pH = 7.4). Material was dehydrated using graded series of ethyl alcohol (10% increase every 15 min), sputtered with gold (50 nm), and observed in scanning electron microscope (EVO LS15, Zeiss) at 10 kV of filament’s tension. 

### 2.10. ROS, SOD, and NO Assays

Kits and reagents were purchased from Thermo Scientific. The level of reactive oxygen species in cells from both tested populations was measured using the 5-(and-6)-chloromethyl-2′,7′-dichlorodihydrofluorescein diacetate reagent (H2DCF-DA). Superoxide dismutase activity was evaluated using SOD assay kit (Life Technologies, San Diego, CA, USA), while the concentration of nitric oxide was measured using the Griess Reagent Kit. All assays were performed in duplicate, according to the instructions provided by the vendor. 

### 2.11. RT-PCR Analysis

Total RNA from cultured cells was isolated with Tri-reagent (Thermo Scientific) and using a phenol chloroform method. Contaminating DNA was digested using DNase I RNase-free (Thermo Scientific) treatment. Reverse transcription was carried out on 100 ng total RNA using Moloney Murine leukemia virus reverse transcriptase (M-MLV RT), provided with the Tetro Reverse Transcriptase kit (Bioline, London, UK). cDNA synthesis were performed following the manufacturers protocol, using a T100 Thermo Cycler (Bio-Rad, Hercules, CA, USA). Quantitative RT-PCR was performed in a total volume of 20 µL using the SensiFast SYBR and Fluorescein Kit (Bioline, London, UK). The PCR reaction profile consisted of initial enzyme activation at 95 °C for 10 s, followed by 40 cycles of denaturation at 95 °C for 15 s, annealing 30 s. with temperature dependent on the primer sequences and extended at 72 °C for 30 s. with a single fluorescence measurement. The series of cycles were followed by a melt curve analysis to ensure reaction specificity. The expression level of each gene was normalized to the housekeeping gene, *GAPDH*. Subsequently, the relative gene expression (*Q*n) was calculated in relation to the *GAPDH* gene.

## 3. Results

### 3.1. Morphology of Films

**Figure 1:**
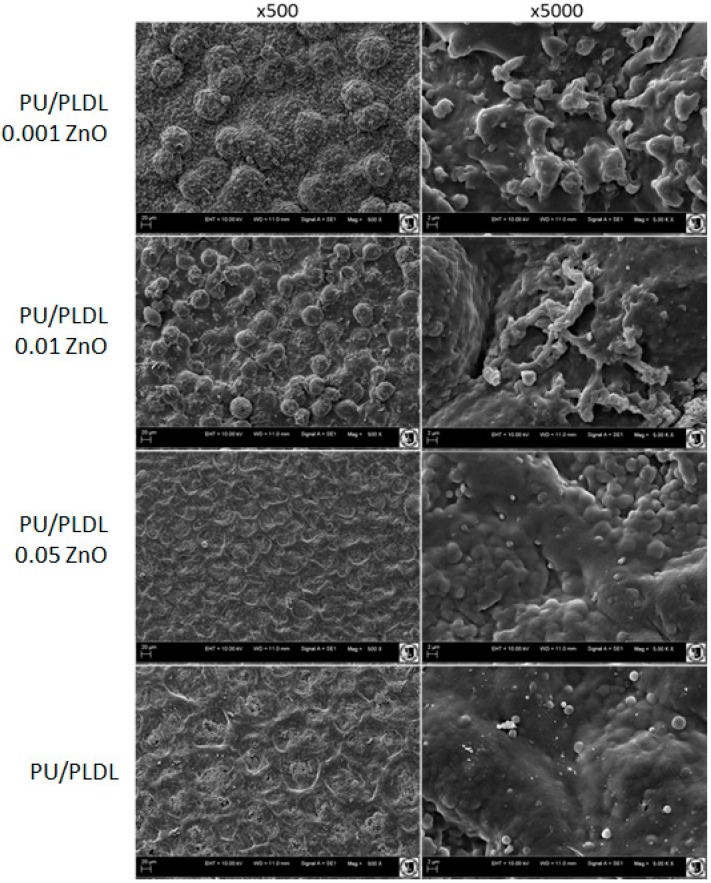
SEM images of PU/PLDL blend doped with different ZnO concentrations. Magnifications 500x (left column) and 5000x (right column).

Observations revealed that there was no prominent difference in the topography of surface between PU/PLDL/0.05 ZnO and pure PU/PLDL control, both at low and higher magnifications. Surfaces were smooth, with shallow, wave-like pattern. In higher magnification the surfaces were less uniform, with visible bubble-like structures (Figure 1). However, the lower concentrations of ZnO added to the polymer blend resulted in more rough surface, with bubbles sized 20–30 µm for 0.01 wt % of ZnO, and 50–70 µm for 0.001 wt % of ZnO. Higher magnifications revealed the presence of irregular structures on the surface of bubble-like elements in both 0.01 and 0.001 ZnO samples (Figure 1).

**Figure 2:**
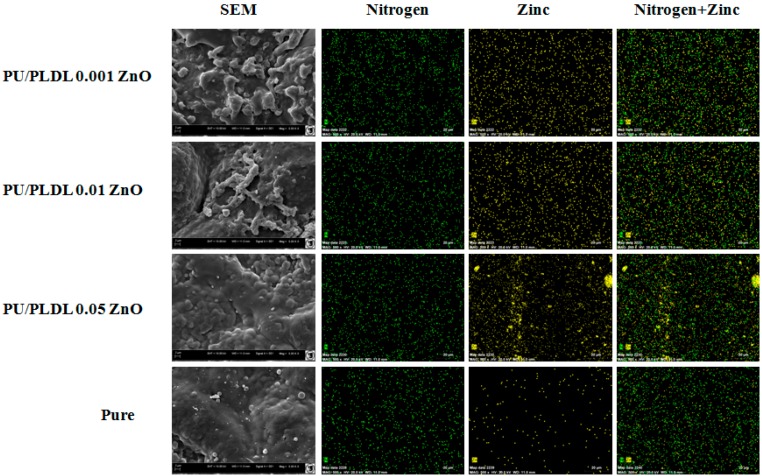
SEM EDX mapping images (nitrogen—green, zinc—yellow) of PU/PLDL/ZnO composites and pure PU/PLDL blend; magnifications 5000× for SEM, 500x for EDX mapping.

The analysis of elemental distribution using SEM-EDX mapping revealed uniform distribution of nitrogen derived from the urethane group in all analyzed samples. In biomaterial doped with the highest concentration of ZnO (PU/PLDL 0.05 ZnO) there were prominent agglomerations of zinc present within the superficial zone of the biomaterial. In biomaterials doped with lower concentrations of ZnO, the distribution of zinc was uniform (Figure 2).

### 3.2. Wettability of Films

The surface wettability of the PU/PLA/ZnO composite films was studied in association with their composition (ZnO concentration). The static water contact angles measured on different polymer composite surfaces are presented in Table 1. The static water contact angle for the pure PU/PLDL blend was previously found to be about 81° [10].

It was found that the PU/PLDL/ZnO composite films had higher water contact angles than pure PU/PLDL blend film at the same measurement conditions, as it is shown in Table 1. Measurements also showed that zinc oxide concentration had significant influence on the surface wettability of PU/PLDL/ZnO composite films. From Table 1, it can be noticed that zinc oxide addition decreased the hydrophilicity of the PU/PLDL blend surface. Surprisingly, with the increase of the amount of zinc oxide, the hydrophobic character of the PU/PLDL/ZnO composites diminished. The most hydrophobic surfaces were obtained for sample PU/PLDL 0.001 ZnO, with a water contact angle of 115.7°.

### 3.3. Atomic Force Microscopy

The ZnO addition significantly alters the PU/PLDL blend surface morphology. The non-doped PU/PLDL film possesses a smother surface with no distinguishable features, while the surface of PU/PLDL/ZnO composite shows a large number of depressions. An overall increase in surface roughness was observed from 400 nm for the PU/PLDL pure blend to 650 nm for the PU/PLDL 0.05 ZnO composite (Table 2).

### 3.4. Proliferation Assay

**Figure 3:**
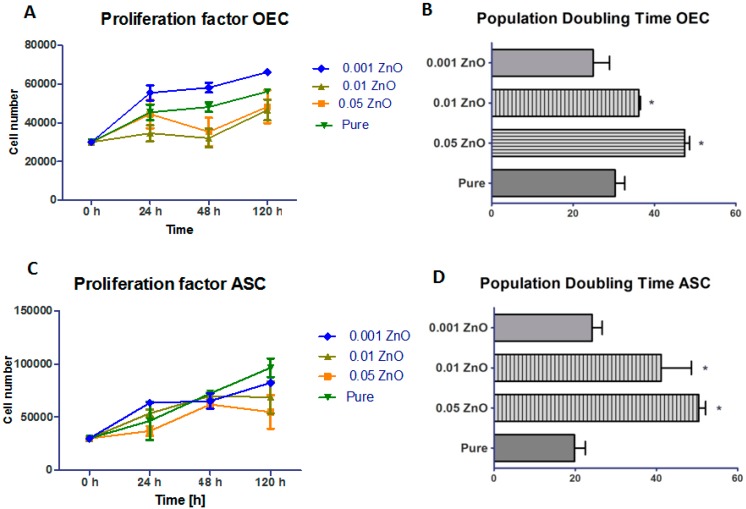
Growth curves (**A**,**C**) and the population doubling times (**B**,**D**) of olfactory ensheathing cells (**A**,**B**) and adipose stem cells (**C**,**D**) during 120 h of experimental culture on different polymers; * *p* < 0.05.

Olfactory ensheathing cells grown on experimental materials showed diverse proliferative activity, regarding the quantity of ZnO present in the polymer. The highest proliferative activity was noticed on sample with the lowest concentration of ZnO (0.001), followed by control sample without the addition of ZnO (pure PU/PLDL), between which the differences in PDT were statistically insignificant (*p* > 0.05). The lowest proliferative activity was observed in sample PU/PLDL 0.01 ZnO (*p* < 0.05), which was similar to results observed in sample PU/PLDL 0.05 ZnO (Figure 3A). The shortest population doubling time was noticed in the sample PU/PLDL 0.001 ZnO and pure PU/PLDL control (*p* > 0.05), while the longest in the sample PU/PLDL 0.05 ZnO (Figure 3B). Adipose stem cells showed a similar response, where the highest proliferative activity was noticed in samples PU/PLDL 0.001 ZnO and pure PU/PLDL control (*p* > 0.05). The lowest proliferative activity was noticed in samples 0.05 and 0.01 (*p* < 0.05) (Figure 3C,D).

### 3.5. Cell Morphology

**Figure 4:**
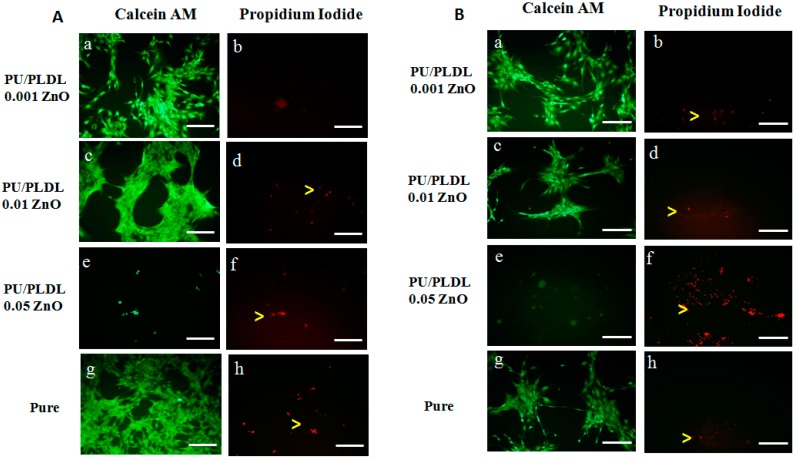
The calcein AM/propidium iodide staining of olfactory ensheathing cells (**A**); and adipose mesenchymal stem cells (**B**) cultured on different polymers; green—live, red—dead (yellow arrowheads); scale bar = 200 µm.

**Figure 5:**
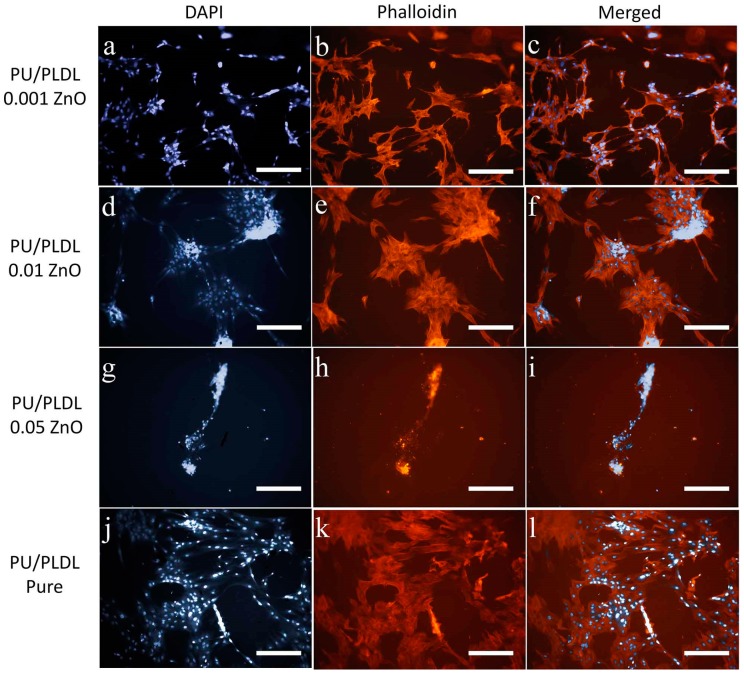
Olfactory ensheathing cells cultured on different experimental polymers stained histochemically for nuclei (DAPI, **a**,**d**,**g**,**j**), and actin (phalloidin, **b**,**e**,**h**,**k**); cell clusters marked with arrowheads; **c**,**f**,**i**,**l**—merged pictures; magnifications 100×, scale bar = 200 µm.

**Figure 6:**
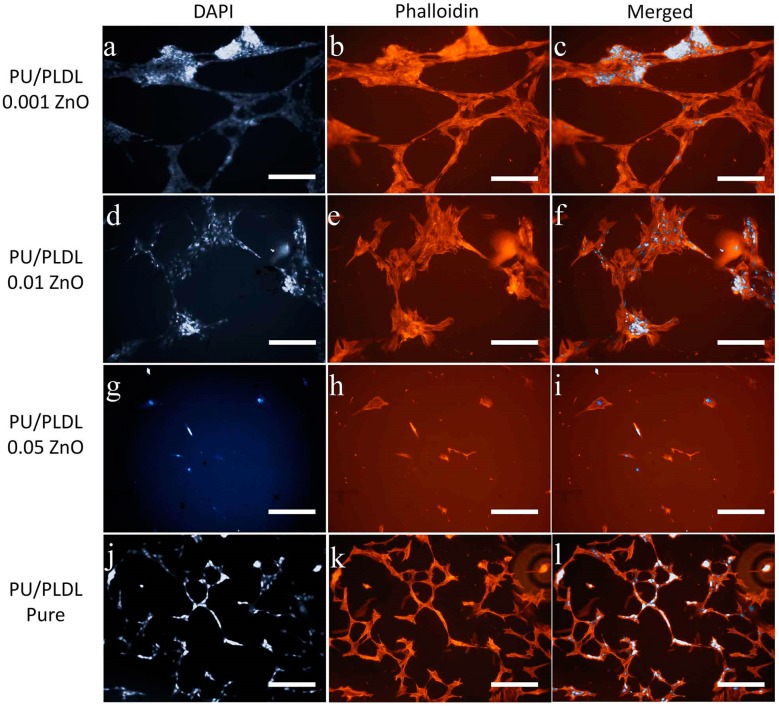
Adipose stem cells cultured on different experimental polymer substrates stained histochemically for nuclei (DAPI, **a**,**e**,**i**,**m**), and actin (phalloidin, **b**,**f**,**j**,**n**), and the cell morphology visualized with SEM (**d**,**h**,**l**,**p**); cell connections marked with arrowheads; magnifications 100× (fluorescence, scale bar = 200 µm).

Olfactory ensheathing cells revealed diverse morphology, regarding the type of material they have colonized. In sample PU/PLDL 0.001ZnO almost all cells were viable and positively stained for calcein AM, and the quantity of dead cells revealed by propidium iodide staining was insignificant. Cells were elongated and created extended colonies connected with each other, with motile cell-type cytoskeleton present. In sample PU/PLDL 0.01 ZnO cells were also mostly calcein-positive, with single dead cells present in the monolayer. Cells were of elongated/tripolar shape and formed clustered colonies connected with each other. Cells cultured on PU/PLDL 0.05 ZnO group were mostly negative for calcein AM, and the propidium iodide staining revealed also the presence of dead cells. Cells were mostly detached from the surface, and the remaining ones created elongated agglomerates. Actin and nuclei staining revealed that cells did not maintain their proper morphology, did not create colonies, and their actin cytoskeleton was significantly reduced. In pure PU/PLDL control cells were mostly viable, and the quantity of dead cells was insignificant. The staining revealed the proper bipolar, elongated shape of OEC and the presence of small, elongated colonies (Figure 4A and Figure 5). Adipose stem cells also showed differences in morphological appearance regarding the type of material they were colonizing. In the sample PU/PLDL 0.001 ZnO and PU/PLDL 0.01 ZnO cells were mostly positive for calcein AM, with only single dead cells present. Actin and nuclei staining revealed that cells had proper, fibroblast-like morphology in these groups. Cells had single nuclei and created irregular clusters. In sample PU/PLDL 0.05 ZnO cells were negative for calcein AM, and a high number of dead cells was detected. Cells were mostly detached from the surface, while only single cells remained on this sample. ASCs cultured on PU/PLDL control were almost all positive for calcein AM and there was no signs of cell death. Cells had single nuclei and properly-developed actin cytoskeleton, without signs of clustering (Figure 4B and Figure 6).

### 3.6. Immunophenotype of OECs

**Figure 7:**
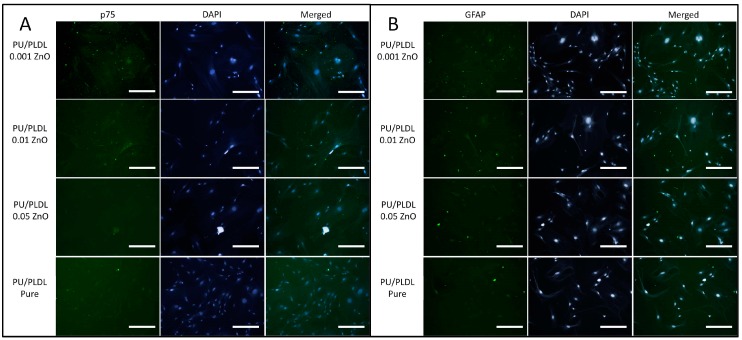
The immunofluorescent staining of olfactory ensheathing cells for specific p75 (**A**) and GFAP (**B**) proteins, combined with nuclei staining (DAPI), magnification 400×, scale bar = 20 µm.

Additional evaluation of specific phenotype of OEC revealed that p75-positive cells were present in all investigated samples, whereas the GFAP-positive cells were not detected in the sample PU/PLDL 0.05 ZnO (Figure 7).

### 3.7. ROS, SOD, and NO Assays

**Figure 8:**
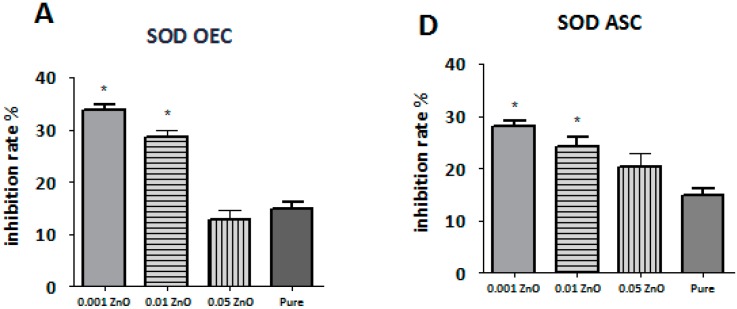

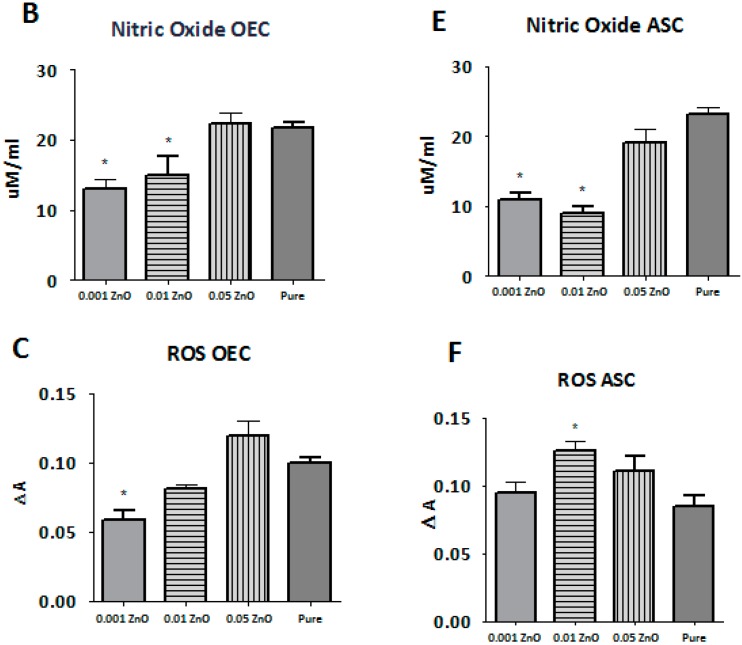
The quantity of superoxide dismutase (**A**,**D**), nitric oxide (**B**,**E**) and reactive oxygen species (**C**,**F**) in olfactory ensheathing cells (**A**,**B**,**C**) and adipose stem cells (**D**,**E**,**F**) cultured on all tested biomaterials (0.001—PU/PLA 80:20 0.001 ZnO, 0.01—PU/PLA 80:20 0.01 ZnO, 0.05—PU/PLA 80:20 0.05 ZnO, Pure—biomaterial without ZnO); * significantly different from control (*p* < 0.05).

The level of ROS detected in olfactory ensheathing cells was different in each investigated sample. The highest level of ROS from samples doped with ZnO was noticed in sample PU/PLDL 0.05 ZnO, which was at the comparable level with pure control (*p* > 0.05). The lowest level of ROS was noticed in sample PU/PLDL 0.001 ZnO (*p* < 0.05). Cells cultured on sample PU/PLDL 0.001 ZnO showed also the highest level of SOD, with its graded decrease in samples with increasing concentration of ZnO. The lowest level of NO in OEC population was noticed in sample PU/PLDL 0.001 ZnO, while the highest was noticed in sample PU/PLDL 0.05 ZnO and pure control (*p* > 0.05) (Figure 8A–C). In adipose stem cells cultured on sample PU/PLDL 0.05 ZnO the level of ROS was increased in relation to pure control, while in the remaining two groups it was decreased (PU/PLDL 0.001 ZnO—*p* < 0.05). Evaluation of NO concentrations in ASC revealed the highest level in pure control, and the lowest in samples 0.01 and 0.001 (*p* < 0.05). Measurements of SOD in the ASC population revealed its highest activity in sample 0.001 and 0.01(*p* < 0.05), while the lowest was noticed in sample 0.05 (Figure 8 D–F).

### 3.8. Real-Time RT-PCR Measurements

**Figure 9:**
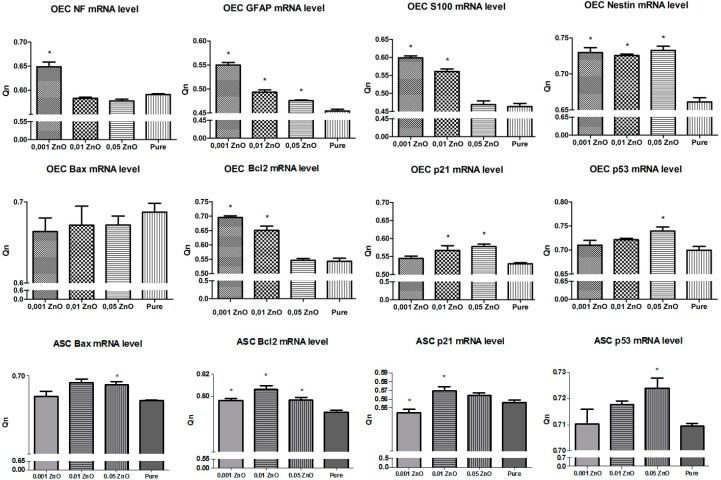
The real-time RT-PCR results obtained from olfactory ensheathing cells (OEC) and adipose mesenchymal stem cells (ASC); * significantly different from control (*p* < 0.05).

In order to determine the *in vitro* physiological status of OECs, we examined expression of *nestin* as neural progenitor cell marker, *S100*, *NF*, and *GFAP* neural stem cell markers. The results of real-time RT-PCR confirmed positive expression of neural markers on biomaterials doped with ZnO. Interestingly, the highest expression of neural markers showed cells cultured on sample PU/PLDL 0.001 ZnO. Having demonstrated that biomaterials doped with higher concentration of ZnO inhibited ASCs and OECs growth, our attention was focused on the potential mechanisms mediating this effect. Our supposition is that the addition of ZnO to the biomaterial promotes apoptosis. To determine the relevance of this pathway in our experiment, we examined the effects of ZnO-doped biomaterials on *p21*, *p53*, *Bax*, *Bcl-2* expression. Both 0.01 and 0.05 ZnO significantly induced *p53* and *p21* mRNA levels in OEC and ASC cells as determined by real-time RT-PCR. In accordance with the changes observed in *p21* and *p53* mRNA, *Bcl-2* and *Bax* genes expression was studied. The higher mRNA copy number for *Bcl-2* was confirmed in OEC and ASC cultured on biomaterials doped with 0.001% ZnO. The *Bax* mRNA copy number was upregulated in all groups, except for OEC and ASC cultured on 0.001% ZnO biomaterial. The higher mRNA copy number for *Bax*, compared to *Bcl-2*, was observed in both OEC and ASC cultured on biomaterial doped with 0.01 and 0.05 ZnO (Figure 9).

## 4. Discussion

Polymer-based biomaterials are being continuously improved both for their higher biocompatibility and for their novel therapeutic strategies. Among the numerous applications of polymers in medicine, the usability of polyurethane–polylactide blends as the transplantable, bioactive cell carriers has been repeatedly shown, considering the regeneration of nervous tissue [9,10,26]. In current research we showed the possibility to additionally enrich the PU/PLDL biomaterial with zinc oxide, which is known for its biological activity and the role it plays in CNS regeneration [27]. Moreover, our results indicated that the ZnO may be used for the modification of physical properties of obtained biomaterial. Interestingly, morphological changes of the PU/PLDL/ZnO films were observed in biomaterials with low concentrations of ZnO used, *i.e.*, 0.001 and 0.01. Despite the presence of bubble-like features on the surface of these two biomaterials, SEM-EDX analysis revealed that there were no significant agglomerations of ZnO particles over these sites. Interestingly, the highest concentration of ZnO (0.05) did not significantly alter the morphology of biomaterials’ surfaces, which were very similar to pure PU/PLDL sample. It corresponds with the lowest difference in water contact angle between the pure control and the 0.05 ZnO group. We suppose, that the unexpected results of water contact angle are derived from changed morphology of obtained biomaterials, including the grain size, their length and density, as was previously described and proposed by Jie Han and colleagues [27].

Therefore, the hydrophilicity is strongly dependent on the surface microtopography [28]. This dependence has been explained in terms of the number of singular pores or surface irregularity observed in the SEM images. This feature might have different consequences during cell culture, as the hydrophilicity of surface determine the adhesion of cells and behavior of stem cells [29].

While the influence of physical properties of biomaterials (like roughness or wettability) on cellular behavior is well known, the proliferative activity of cells cultured on experimental polymers was influenced by the presence of ZnO molecules rather than by materials’ physical features—while there were differences in roughness and wettability observed between pure PU/PLDL and 0.001 ZnO, the differences in proliferation between these two groups were not statistically significant. Moreover, while the increase of surface roughness results in increased cell proliferation [30], as it was shown previously, the surface of the highest level of roughness (0.05 ZnO) should improve cell proliferation, whilst we observed the opposite situation. Considering the fact that the increase of ZnO content in polymers resulted in decreased proliferative activity, the highest influence on cell proliferation was derived from the presence of ZnO molecules. 

In addition to antimicrobial activity, zinc has also strong antioxidant and anti-inflammatory properties. Zinc oxide has already been used as a component of various biomaterials [31,32]. In CNS injuries, the oxidative stress plays major role in neuron death due to apoptosis, therefore the use of ZnO-doped biomaterial for CNS repair may reduce this feature. Our results indicated that the lowest concentration of ZnO reduced the levels of reactive oxygen species (ROS) and NO, and increased the activity of superoxide dismutase (SOD) in both tested cell populations. Moreover, the RT-PCR measurements indicated that in the 0.001 group the expression levels of OEC-specific genes (*GFAP*, *S100*, *NF*, and *nestin*) were upregulated, indicating the optimal concentration of ZnO used in biomaterial for tested glial cell population. There was also a significant increase in *Bcl-2* gene expression level in 0.001 and 0.01 groups, while the *p53* and *p21* genes were upregulated in samples with the highest ZnO content (0.01, 0.05). Therefore, it may be suspected that *Bcl-2* exhibited anti-apoptotic effect, while this gene is involved in both the induction and the inhibition of the apoptosis [33]. In the 0.001 group the proliferative activity of olfactory ensheathing cells and adipose stromal cells was at the highest level. Moreover, the morphology and phenotype of cells was not altered in this group, while cells cultured on biomaterials doped with 0.01 and 0.05 ZnO were more aggregated, probably by the changes in surface hydrophilicity. The highest concentration of ZnO used in polymers exerted the negative effect on tested cells, resulting in decreased proliferation, alterations in morphology and phenotype, increased apoptosis and the level of oxidative stress, which is consistent with previous observations. The other studies indicated that ZnO may induce the cytotoxic effects in cells at certain concentrations or form, such as nanoparticles [34,35,36,37]. It should be underlined however, that substances known as safe for cells, may exert cytotoxic effect when they form nanoparticles [38]. Yet the presence of zinc oxide nanoparticles on PU/PLDL ZnO biomaterials is currently investigated in our present research. Despite the fact that the 0.001 ZnO group showed even lower proliferative activity than pure PU/PLDL, the advantage of ZnO addition is connected with its anti-apoptotic and anti-oxidative stress activity. Thus, even while the pure PU/PLDL and 0.001 ZnO groups showed the similar cell density, cell physiological status was improved in 0.001 ZnO, concerning the downregulation of proapoptotic genes and decrease in ROS, NO concentrations, and increase in SOD activity.

Zinc plays also important role in the development and homeostasis of central nervous system, as it has been found in certain cerebrocortical regions of brain, including the hippocampus known for its neoneurogenesis [18,19,39]. The positive cellular response obtained from olfactory ensheathing glial cells, which have been considered as promising tool in CNS regeneration, indicated on the desired properties of zinc-enriched PU/PLDL designed as a cell carrier for transplantation. Increase in the proliferation of ASC cultured on ZnO-enriched films indicated on the possibility to use this biomaterial as mesenchymal stromal cell carrier in current and future therapies, while the ZnO present in the biomaterial decreases the risk of microbial contamination and additionally reduces the inflammatory response expected after the transplantation.

## 5. Conclusions

The polyurethane/polylactide blends have been previously found as a biocompatible material designed for regenerative medicine applications. In this study the blend consisting of 80/20 *w*/*w* PU/PLDL has been enriched with zinc oxide nanopowder. The addition of 0.001% of zinc oxide to PU/PLDL films exerted *in vitro* positive influence on both olfactory ensheathing glial cells and adipose stromal cells cultured on biomaterials, increasing their viability and proliferative potential, while decreasing the oxidative stress and the level of apoptosis. Thus, a polyurethane-polylactide (80:20) blend doped with 0.001% of zinc oxide may be considered as a smart, biocompatible, and bioactive cell carrier which supports cell recovery and the process of regeneration.

## Figures and Tables

**Table 1:** Water contact angle measurements of PU/PLDL blend cast films doped with different amounts of zinc oxide.

Sample	Water contact angle (°)
PU/PLDL (control)	81.3 ± 0.9
PU/PLDL 0.05 ZnO	95.7 ± 4.5
PU/PLDL 0.01 ZnO	101.3 ± 5.8
PU/PLDL 0.001 ZnO	115.7 ± 6.3

**Table 2:** Surface roughness values (*R*a) of PU/PLDL blend and PU/PLDL/ZnO composite obtained using AFM analysis.

Sample code	*R*q (nm)	
	Top side	Bottom side
**PU/PLDL (control)**	409 ± 65	198 ± 78
**PU/PLDL 0.001 ZnO**	575 ± 97	248 ± 56
**PU/PLDL 0.01 ZnO**	613 ± 85	267 ± 23
**PU/PLDL 0.05 ZnO**	747 ± 109	319 ± 92

## Abbreviations

The following abbreviations are used in this manuscript:

CNS	Central nervous system
SCI	Spinal cord injury
PU	Polyurethane
PLDL	Poly(l-lactide-*co*-d,l-lactide)
OECs	Olfactory ensheathing cells
ASCs	Adipose stromal stem cells
ROS	Reactive oxygen species
NO	Nitric oxide
SOD	Superoxide dismutase
FBS	Fetal bovine serum
DMEM	Dulbecco’s Modified Eagle Medium
HBSS	Hank’s Balanced Salt Solution
GFAP	Glial fibrillary acidic protein
